# Supplementary material for: Electrospun Silicon Dioxide/poly(vinylidene fluoride) Nanofibrous Membrane Comprising a Skin Multicore–Shell Nanostructure as a New High-Heat-Resistant Separator for Lithium-Ion Polymer Batteries
Source: Polymers (Basel). 2024 Jun 26;16(13):1810. doi: 10.3390/polym16131810 (PMC11244255; doi:10.3390/polym16131810)
Supplement: Supplementary file 1 [file polymers-16-01810-s001.zip › polymers-3007484-supplementary.pdf]

Supplementary Data

1. Experimental

Prior to the test, a proto-type cell is fully charged with cut-off voltages of 2.75 and 4.2 V at a 1.0 C-rate in the constant current (CC) mode and the constant voltage (CV) mode. After resting for 10 minutes, the cell is discharged under the constant current mode (1.0 C-rate) for 6 minutes to deliver a state of 10% depth-of-discharge (DOD), which also means 90% of SOC. Next, the test is conducted as follows: discharging at the rate of 5.0 C for 10 seconds, resting for 40 seconds and charging at the rate of 3.0 C for 10 seconds to determine the 10-second discharge-pulse and 10-second regeneration-pulse power capabilities at each 10% DOD increment. This test is separated by 10% DOD constant current discharge segments; each 10% DOD segment has a 20 minutes rest period that leads the cell bring to electrochemical and thermal equilibrium before the next profile. The same steps are repeated as shown above until 90% of DOD is reached.

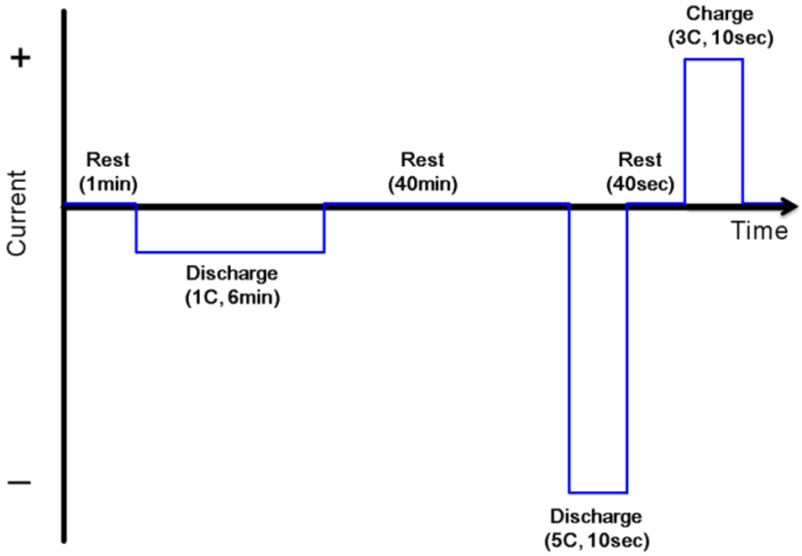

Figure S1. Sequence for test of the HPPC[1, 2].

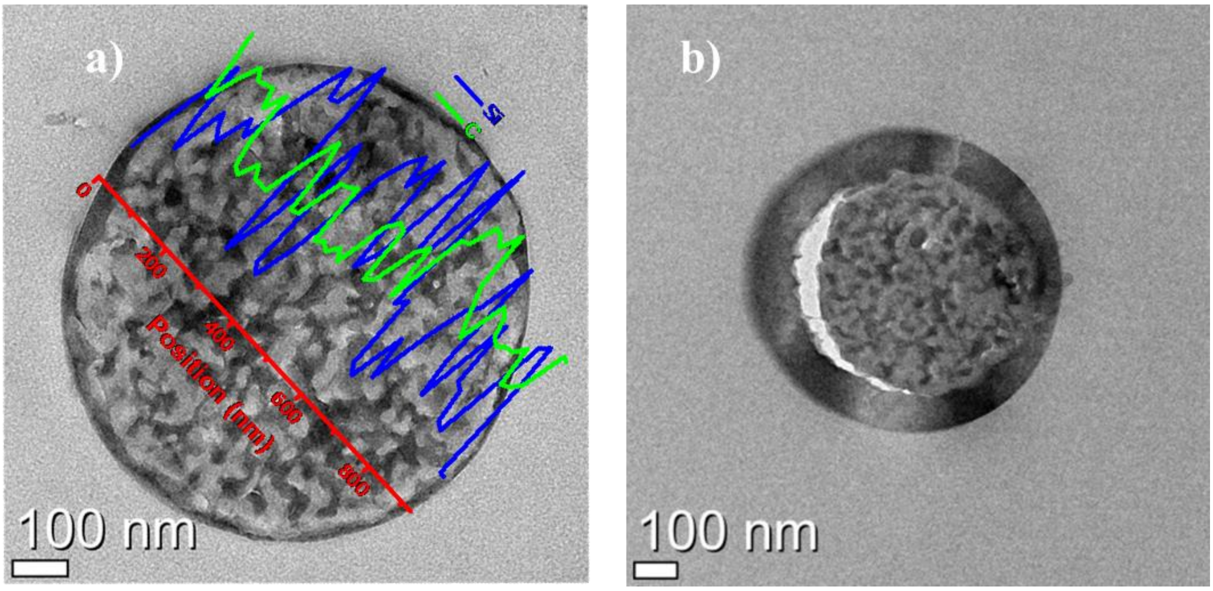

Figure S2. Cross-sectional TEM Images of SiO_2-PAN electrospun fiber. Specimen used in TEM analysis was prepared by using microtome method under cryo- condition. a) bright field image of SiO_2-PAN = 5:5 (wt.ratio) and elemental line profile along the red line depicted in image; and b) bright field image of SiO_2-PAN = 6:4 (wt. ratio).

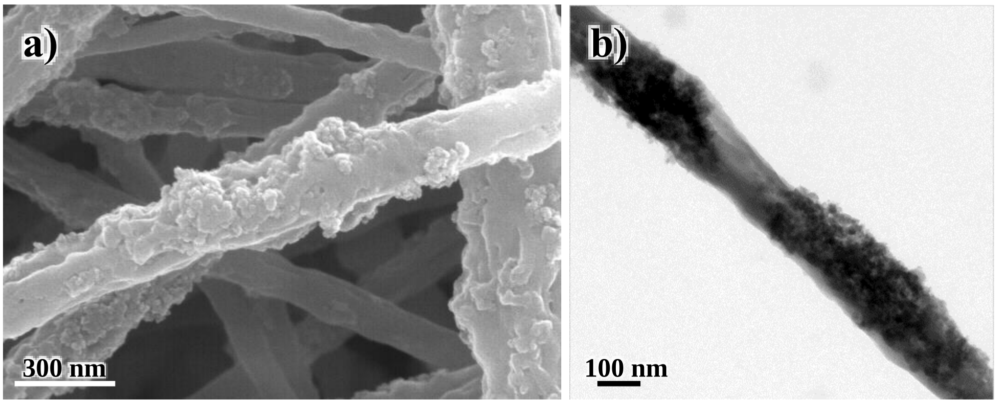

Figure S3. SEM and TEM images of electrospun fumed silica-PVdF (=3:7 wt.ratio) blend nanofiber. a) SEM image; and b) bright field image without ultramicrotome

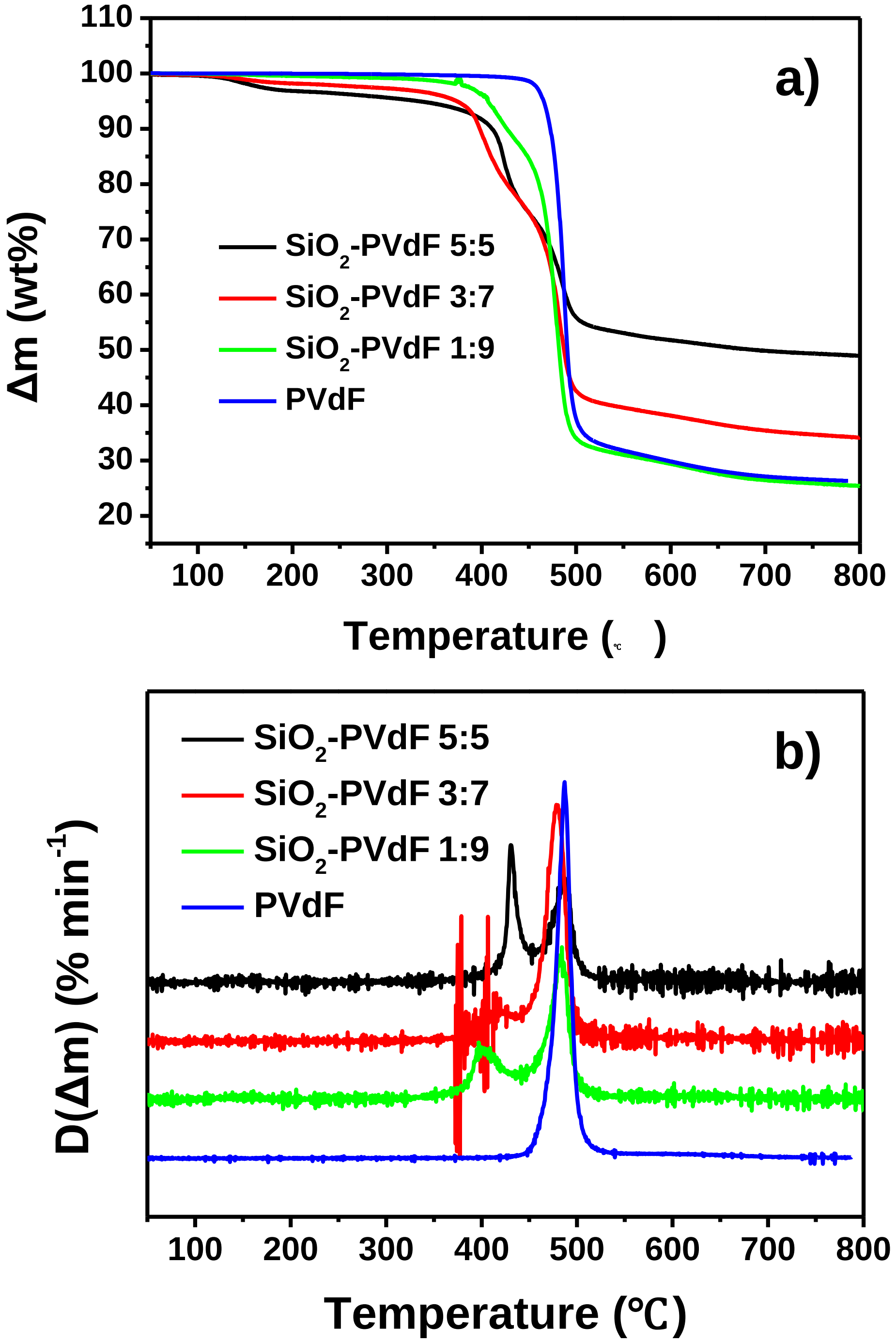

Figure S4. Thermogravimetric analysis (10 °C min⁻¹) of pure PVDF and as-prepared SiO_2-PVDF electrospun membrane under the nitrogen flow. a) Thermogravimetry and b) derivative thermogravimetry curves.

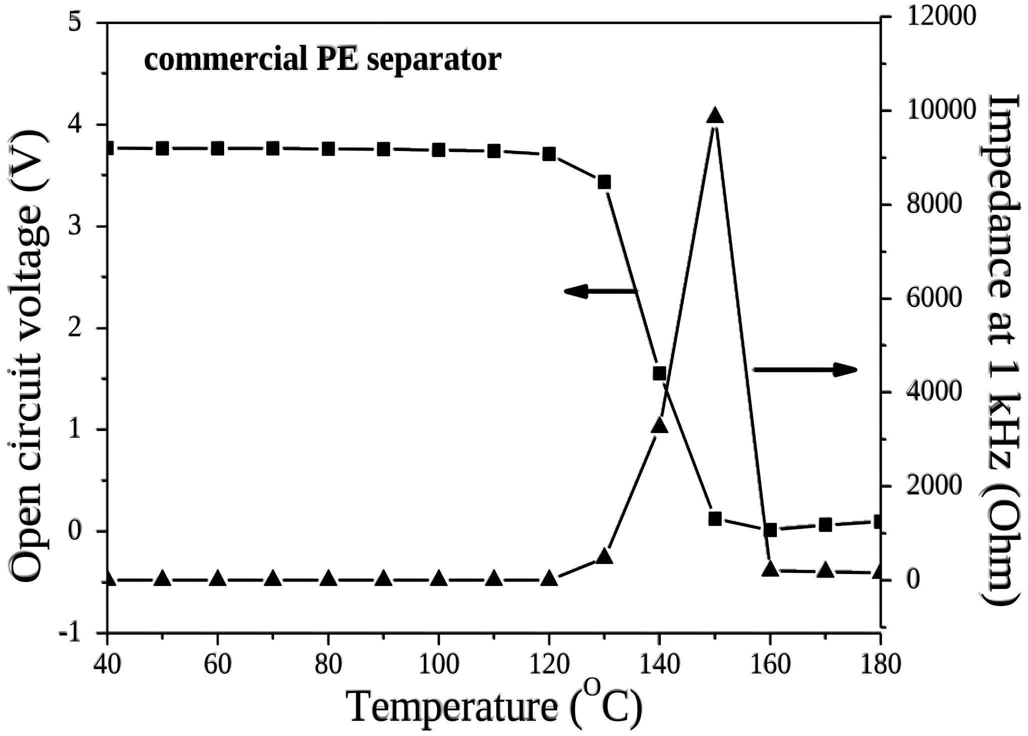

Figure S5. Thermal stability of the proto-type cell with PE separator containing 1M $LiPF_6$-EC/PC/DEC/VC.

Reference

(1) Energy, U. S. D. o. *PNGV(Partnership for a new generation of vehicles) Battery Test Manual. Rev.3*; DOE/ID-10597; Avenue Southwest, Washington, D.C., U.S., 2001.
(2) Laboratory, I. N. *Battery Test Manual for Plug-In Hybrid Electric Vehicles*; INL/EXT-07-12536; Idaho Falls, Idaho, U.S, 2010.
